# Chromosomal instability and its effect on cell lines

**DOI:** 10.1002/cnr2.1822

**Published:** 2023-04-24

**Authors:** Zichen He, Andrew Wilson, Fenella Rich, Diane Kenwright, Aaron Stevens, Yee Syuen Low, Michelle Thunders

**Affiliations:** ^1^ Department of Pathology and Molecular Medicine University of Otago Wellington New Zealand

**Keywords:** cell culture, cell lines, chromosome instability, mitotic checkpoint, telomere

## Abstract

**Background:**

Cancer cell lines are invaluable model systems for biomedical research because they provide an almost unlimited supply of biological materials. However, there is considerable skepticism regarding the reproducibility of data derived from these in vitro models.

**Recent findings:**

Chromosomal instability (CIN) is one of the primary issues associated with cell lines, which can cause genetic heterogeneity and unstable cell properties within a cell population. Many of these problems can be avoided with some precautions. Here we review the underlying causes of CIN, including merotelic attachment, telomere dysfunction, DNA damage response defects, mitotic checkpoint defects and cell cycle disturbances.

**Conclusion:**

In this review we summarize studies highlighting the consequences of CIN in various cell lines and provide suggestions on monitoring and controlling CIN during cell culture.

## 1 INTRODUCTION

Cell lines are clonally multiplied cells that can be propagated repeatedly, and may have an indefinite lifespan if they have been immortalized.^1^ This potential for unlimited growth makes them critical resources for biomedical research because of the limited availability of human tissues. Many revolutionary milestones in biology and modern medicine have been closely related to the establishment of cell lines. For example, the development of the first human polio vaccine was facilitated by using the first and most popular human cell line, the HeLa cell line.^2^ Cell lines offer many advantages as biological models, such as affordability, indefinite proliferative ability, and ease of manipulation. Also, the use of established cell lines bypasses complicated ethical issues involved in animal and human experiments. As of today, thousands of cell lines have been isolated from all types of normal and diseased human tissues and are widely used by the research community worldwide. It was estimated that cell lines have contributed to approximately 2 million publications by the year 2018.^3^

As biological model systems, cell lines are assumed to maintain a relatively stable genomic landscape that can represent the tissue of origin. However, this assumption has been disputed by a growing body of research, in which genetic instability has been explicitly characterized in various cell lines during in vitro culture.^4^, ^5^, ^6^ Over time, cell lines can undergo considerable genetic and phenotypic changes and deviate significantly from their original functional features. As such, unstable cell lines would not be able to represent the cells from which they were derived. While this does not necessarily mean that the research conducted with these cells is invalid, it can unconsciously tamper with the data and produce inconsistent, irreproducible results.

Genomic instability, one of the enabling characteristics of cancer,^7^ is a significant problem that affects the utility of cell lines. Cancer cell lines are especially prone to genetic variations due to the presence of mutations in critical oncogenes and tumor suppressor genes, such as *TP53, PTEN*, and *KRAS*.^8^ Chromosomal instability (CIN) is the most prevalent form of genomic instability, occurring in around 80% of tumors.^9^ CIN describes an active state in which chromosome mis‐segregation occurs at a persistently high rate, characterized by ongoing changes in chromosome number and structure.^10^ Generally, numerical CIN involves changes in the number of intact chromosomes, referred to as whole‐chromosome aneuploidy; structural CIN is characterized by gross chromosomal rearrangements, mainly including deletions, amplifications, inversions and translocations. During in vitro culture, some of these chromosomal aberrations may contribute to the development or maintenance of the cancer phenotype, while the majority of them are not selected for and eventually get eliminated.^11^ CIN in cell lines is often followed by phenotypic drift when some variants are selected over the original cell populations, and this process tends to progress with increasing passages.^12^

In a research setting, CIN is often a consequence of CRISPR‐Cas9 gene editing. One of the major concerns associated with the use of CRISPR‐Cas9 is the potential for unintended off‐target effects.^13^ The guide RNA used in CRISPR‐Cas9 is designed to target a specific DNA sequence, but it is possible that the guide RNA may also bind to and cleave other DNA sequences that are similar in sequence, leading to unintended mutations or genetic changes. CIN can occur when the double‐strand breaks (DSBs) created by CRISPR‐Cas9 are not properly repaired by the cell's DNA repair mechanisms.^14^ This can lead to the loss of large regions of DNA or large‐scale chromosomal rearrangements, which can have serious consequences for the cell.^15^, ^16^

CIN poses a significant challenge to cancer research because the genetic and phenotypic heterogeneity that arises due to CIN in cell lines can compromise the accuracy and reproducibility of experiments. Additionally, the genetic changes that occur in cell lines over time may not accurately reflect the genetic alterations present in the original tumor. While background controls are often included in published studies, they may not fully capture the indirect effects of CIN. For instance, Chromosome segregation errors during mitosis can produce unstable micronuclei that rupture and release genomic DNA into the cytosol.^17^ The cGAS–STING (cyclic GMP‐AMP synthase–stimulator of interferon genes) cytosolic DNA‐sensing pathway recognizes the double‐stranded DNA in the cytosol and initiates an inflammatory response. Chronic activation of the cGAS‐STING pathway promotes cancer invasion and metastasis through STING and downstream noncanonical NF‐κB pathway.^18^ Another consequence of CIN is the formation of extrachromosomal DNA (ecDNA) hubs, which can lead to the rapid malignant transformation of cells.^19^ ecDNAs are circular DNA fragments that can harbor oncogenes, and their amplification can result in their overexpression, leading to cancer initiation and progression.^19^, ^20^ Such mechanisms further complicate experimental interpretation and limit the translational potential of preclinical models.

This review will provide an overview of the fundamental mechanisms of CIN, highlight the implications of CIN in fundamental research, and discuss how CIN can be monitored and controlled during cell culture.

## 2 MECHANISMS OF CIN

**FIGURE 1:**
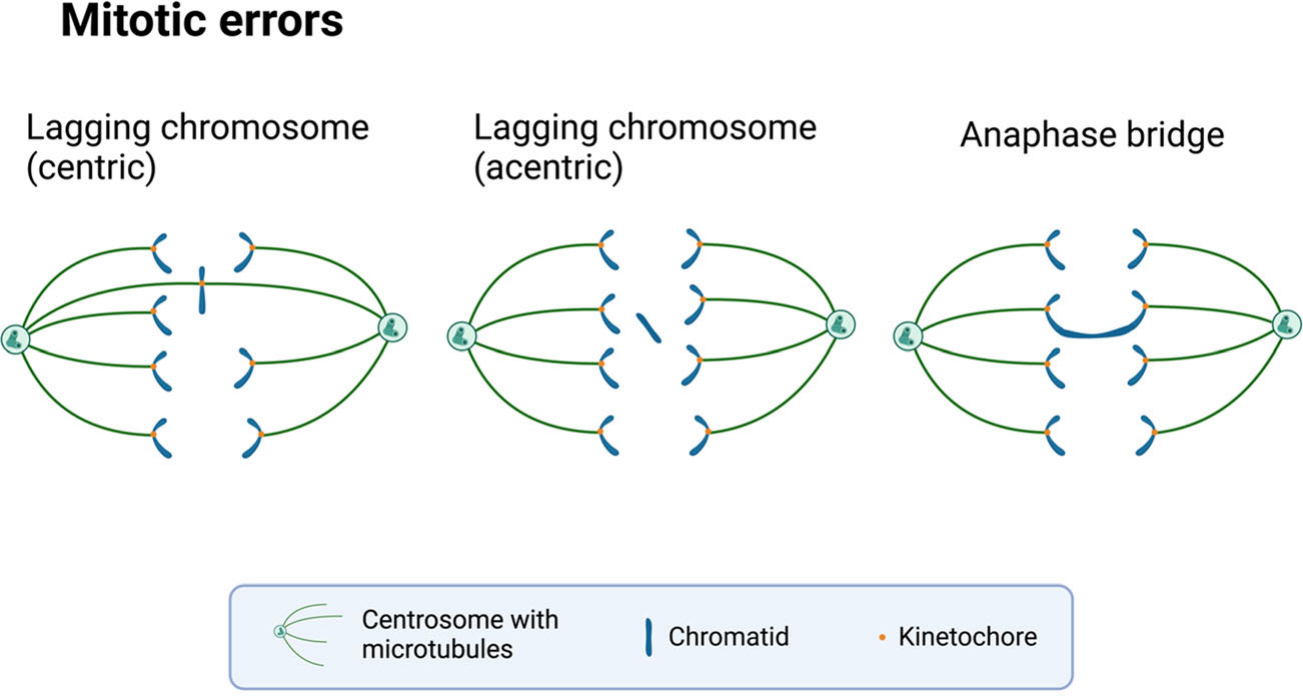
Three major forms of mitotic errors. Lagging centric chromosomes are whole chromosomes lagging in the spindle midzone. Lagging acentric fragments are partial chromosomes that lack centromeric signals. Anaphase bridges may lead to chromosomal breakage and structurally abnormal chromosomes.

Chromosome stability relies on the correct separation of duplicated chromosomes to daughter cells during mitosis.^21^ However, mitosis is a highly complex process. Errors in any part of this process, including cell division machinery, mitotic checkpoints, and gene repair systems, can render the chromosomes unstable and lead to numerical and structural chromosomal abnormalities. Defective chromosome segregation during anaphase spawns three forms of visible mitotic errors: lagging chromosomes, acentric chromatin fragments, and anaphase chromatin bridges (Figure 1).^21^ Lagging chromosomes are characteristic of numerical chromosomal instability, whereas acentric chromatin fragments and anaphase bridges are characteristic of structural chromosomal instability.^22^

Over the past two decades, extensive work has been dedicated to understanding the molecular principles underlying CIN. To date, numerous processes that contribute to CIN have been identified, including defects in kinetochore‐microtubule (k‐MT) attachment, telomere maintenance, DNA damage response (DDR), mitotic checkpoints and cell cycle regulation. While these mechanisms have varied prevalence across different cell types and were assumed to be independent, they are intrinsically connected and often interact with each other.

## 3 MEROTELIC ATTACHMENT

**FIGURE 2:**
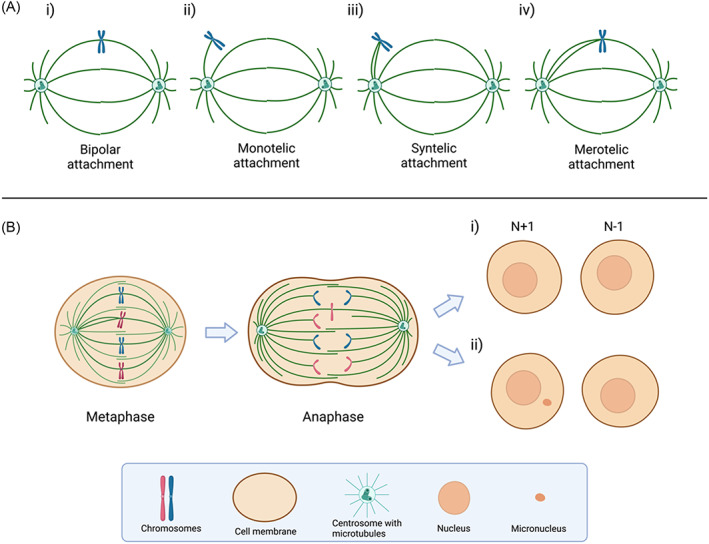
Abnormal kinetochore‐microtubule attachments. (A) Types of kinetochore‐microtubule attachment. (i) Bipolar attachment. (ii) Monotelic attachment. (iii) Syntelic attachment. (iv) Merotelic attachment. (B) Consequences of merotelic attachment. (i) Whole‐chromosome aneuploidy. (ii) Formation of micronuclei.

Chromosome segregation is the fundamental process of cell division; fidelity in this process during mitosis is crucial for genomic stability. Correct bipolar attachment is a prerequisite for proper chromosome segregation, which demands that kinetochores on every sister chromatid pair attach to microtubules emanating from opposite centrosomes (Figure 2Ai). There are three types of abnormal k‐MT attachments: monotelic (Figure 2Aii), syntelic (Figure 2Aiii) and merotelic (Figure 2Aiv).^23^ Monotelic and syntelic attachments can activate the mitotic checkpoint, suspending chromosome segregation until all chromosomes are properly attached to spindle microtubules via their kinetochores. In contrast, merotelic attachment cannot be sensed by the mitotic checkpoint because kinetochores on both sister chromatids are theoretically occupied by microtubules, generating tension needed to silence the checkpoint mechanism.^23^ During chromosome segregation, chromosomes with merotelic attachments are often left behind at the spindle equator, giving rise to lagging chromosomes. Some lagging chromosomes may eventually segregate to the opposite pole and lead to whole‐chromosome aneuploidy (Figure 2Bi). If a lagging chromosome fails to segregate to one of the daughter nuclei before the nuclear membrane is reformed, it will be isolated and form a micronucleus (Figure 2Bii).^24^

## 4 TELOMERE DYSFUNCTION

**FIGURE 3:**
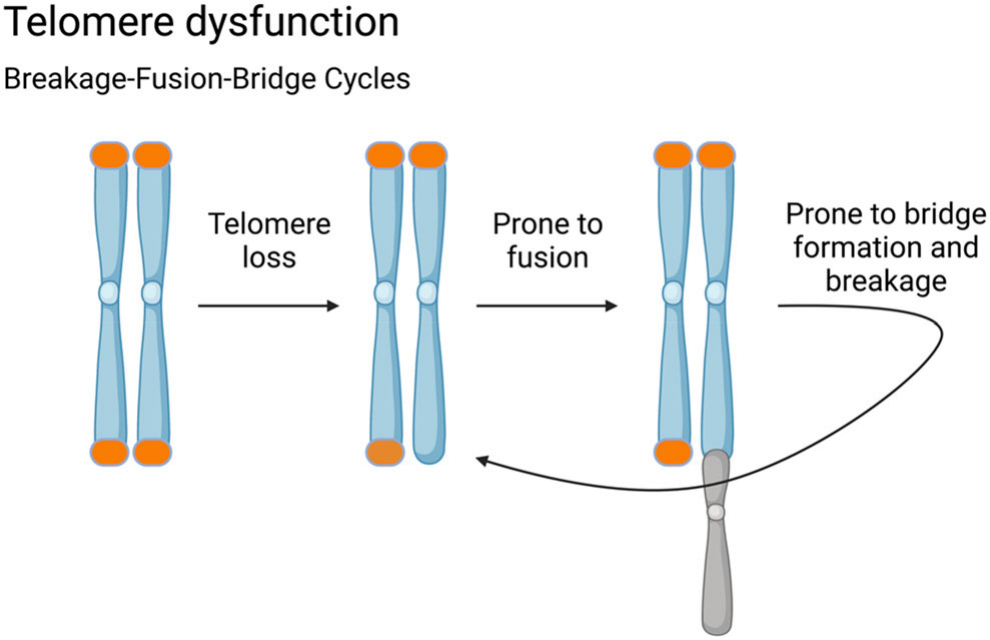
Telomere dysfunction leading to breakage‐fusion‐bridge cycles.

Another mechanism that has been proposed to induce CIN is defective telomere maintenance. Telomeres are regions of repetitive DNA sequences found at the ends of chromosomes that protect the genetic material from degradation or fusion with other chromosomes.^25^ Telomeres are essential for maintaining chromosome stability and integrity during cell division. As cells divide, the telomeres gradually shorten, which can eventually lead to cellular aging or dysfunction.^25^, ^26^ In cases where cells escape this mechanism, uncapped chromosomes can be recognized as DNA breaks and are prone to end‐to‐end chromosome fusion that gives rise to ring or dicentric chromosomes.^27^ Such chromosome abnormalities can initiate CIN through a process called breakage–fusion–bridge cycles. This process involves the formation of bridges by dicentric chromosomes that break when the cell attempts to divide, and the cycle repeats itself in subsequent cell cycles (Figure 3).^25^ One common feature of immortalized cell lines is that they have artificial amplification of telomerase reverse transcriptase, leading to artificially lengthened telomeres.^28^

## 5 DNA DAMAGE RESPONSE DEFECTS

Chromosomal DNA is constantly damaged by internal and external factors, such as ionizing radiation, ultraviolet radiation, and reactive oxygen species.^29^ Depending on the nature of the damage, DNA can be altered in many different ways spanning from nucleotide alterations to DNA strand breaks.^30^, ^31^ To preserve genomic integrity, human cells have a robust DDR mechanism that can pause cell‐cycle progression and activate DNA repair machinery. Several mechanisms for repairing specific types of DNA damage have been identified. These include non‐homologous end joining (NHEJ) and homologous recombination (HR) for repairing DSBs,^32^ single‐strand break repair (SSBR) for ligating broken DNA strands,^33^ mismatch repair (MMR) for correcting replication errors,^34^ base excision repair (BER) for reversing oxidative base modifications,^35^ and nucleotide excision repair (NER) for removing lesions that distort the DNA helix.^36^ When the DNA damage is correctly repaired by these mechanisms, DDR is inactivated and the cell resumes its normal functions. However, if the DNA lesions cannot be fully repaired, prolonged DNA damage can lead to chromosome segregation defects and subsequent CIN.^22^, ^37^

A novel connection between DDR and CIN was discovered by Bakhoum et al.^22^ They found that when DNA damage frequently occurs during mitosis, DDR can be partially activated and causes unanticipated chromosome mis‐segregation and aneuploidy. This is because DDR‐related proteins, activated by DNA damage during mitosis, excessively stabilize k‐MT attachments through Aurora A and Polo‐like kinase 1, thereby enabling the production of lagging chromosomes during anaphase.

## 6 MITOTIC CHECKPOINT DEFECTS

The mitotic checkpoint, also known as the spindle assembly checkpoint (SAC), is a critical surveillance mechanism that maintains the correct chromosome number during mitosis.^38^ Proper chromosome segregation requires sister chromatid cohesion to be maintained through the G2 and M phases of the cell cycle before being abruptly disrupted at the beginning of anaphase; the SAC is responsible for controlling the precise timing of the disruption of the cohesion complex.^39^

The key components of the SAC include Mitotic arrest deficiency 1 (Mad1), Mad2, budding uninhibited by benzimidazoles 1 (Bub1), Bub3, BubR1, and Cell division cycle protein 20 homolog (Cdc20).^40^ These components generate the checkpoint signal that delays anaphase onset and preserves chromatid cohesion until all chromosomes are correctly attached to spindle microtubules. Faulty SAC allows mitosis to proceed despite an aberrant chromosomal structure at metaphase and considerably increases the possibility of chromosome mis‐segregation.^41^ Impairment of specific SAC components has been identified to cause aneuploidy and CIN. For example, mutations in the gene encoding BubR1, a key SAC protein, can lead to a rare disorder known as mosaic variegated aneuploidy, which is characterized by mosaic aneuploidy due to increased chromosome mis‐segregation.^42^

## 7 CELL CYCLE DISTURBANCES

The mechanism of cell cycle disturbances is a broad topic because it is implicated in many of the mechanisms mentioned above. There are many cell‐cycle regulators that play an important role in CIN, such as cyclin E, p53 and forkhead box protein M1 (FoxM1).^39^ It has been elucidated that these proteins are connected to CIN via three general pathways. First, some cell‐cycle regulators may directly maintain chromosomal segregation fidelity during mitosis. Second, dysregulation of some cell‐cycle regulators outside of M phase may indirectly facilitate chromosome mis‐segregation. Finally, disruption of cell‐cycle checkpoint proteins may allow cells with mis‐segregated chromosomes to advance through the cell cycle.^40^, ^43^

## 8 CONSEQUENCES OF CIN IN CELL LINES

Established cell lines have played an essential part in biomedical research as an inexhaustible source of biological material. However, researchers have generally found considerable CIN in many different cell lines, and questions have been raised about the comparability of cell lines to the original cells from which they were derived. Previous studies have shown that cell cultures have the potential to undergo major karyotypic changes over time due to CIN, which results in the constant emergence of variants and subsequent heterogeneity within an assumed monoclonal cell line.^5^, ^44^, ^45^ As cells continue to proliferate and are passaged, cell line evolution may occur due to selection pressure. Specific subpopulations with growth advantages will gradually overgrow the original population and become dominant, leading to significant changes in the characteristics of the cells, such as morphology, proliferation rate, transcriptomic profile, and drug sensitivity.^46^, ^47^ Moreover, heterogeneous subclones may lead to inconsistent results when they are used in similar assays.

Vargas‐Rondón^2^ evaluated the cytotoxic effects of chemotherapeutics on breast cancer cell lines with varying levels of CIN and clonal heterogeneity. They found that breast cancer cell lines with higher levels of CIN and clonal heterogeneity had decreased sensitivity to chemotherapy drugs, suggesting that CIN and clonal heterogeneity may play a significant role in drug resistance and treatment failure in breast cancer. Tripathi et al.^48^ suggest that aneuploidy contributes to immune evasion in cancer by suppressing tumor antigen presentation. They found that aneuploid cancer cells had reduced expression of genes involved in antigen presentation, which led to decreased recognition and killing of cancer cells by immune cells, thus a potential mechanism through which cancer cells are able to evade immune surveillance and promote tumor progression. The findings may provide new targets for cancer immunotherapy. These are two examples of from a large amount of literature attempting to investigate the implications of CIN in cultured cell lines. Two strategies are commonly used; some studies compare cells of early and late passages to demonstrate the deviations in genetic and transcriptional profiles, alternatively, randomly selected subclones are compared to uncover cellular heterogeneity within a common parental cell line. Table 1 summarizes some significant studies that highlighted the phenotypic and functional consequences of CIN in cell lines.

**TABLE 1:** A summary of studies investigating functional consequences of CIN.

Cell line	Comparison	Consequences	Reference
Jurkat	Subclones	Observation of the parent Jurkat line and four subclones for a period of 2 years revealed that one subline exhibited a faster growth rate compared to the other sublines and the parental line. Karyotypic variability was observed within 2 months of cloning and tended to increase with time. Each subline had a distinct morphology and protein expression profile after 40 weeks of culture	[49]
MCF7	Subclones	There is significant genetic variation across 27 MCF7 strains, with only a small minority of genetic changes (13% of gains and 21% of losses) being detected in all strains. Ten chromosome arms (25% of the genome) were differentially gained or lost in a pairwise comparison of strains. The researchers detected 283 genes with copy number gains and 405 genes with copy number losses (compared to basal ploidy) in at least one strain	[50]
HeLa	Subclones	A comparison of the DNA content between HeLa cells and the diploid human genome revealed a significant variation among the four subclones. Different HeLa clones displayed differences in gene expression in response to a hypoxic stimulus. New specific markers were identified in each clone	[45]
RAW 264.7	Passages	The phenotype (expression of macrophage‐characteristic genes and markers) and functional characteristics (phagocytosis activity and NO production) of RAW 264.7 cell line remain relatively stable from passage 10 up to passage 30, but get increasingly unstable afterwards. The expression levels of *CD86*, *HIF‐1α*, *CD11a*, *CD18*, *CD206*, *CD200R*, *Glut1* (Glucose transporter 1), and *Ly6c* increased consistently from passage number 5 up to passage number 50. The expression of *CD11b* was notably reduced in older passages. The expression of *F4/80*, which is responsible for regulating cell adhesion and the induction of CD8+ lymphocyte, remained constant up to passage number 50	[4]
SAOS	Passages	High‐passage Saos‐2 cells demonstrated a higher rate of proliferation, a lower level of alkaline phosphatase activity, and a significant reduction in the expression of decorin	[51]
	Passages	The SAOS cells showed altered gene expression with increasing passage number. 810 genes were up‐regulated (>2‐fold) while 487 genes were down‐regulated in late vs. early passages. Genes involved in hedgehog and WNT signaling pathways were significantly up‐regulated	[47]
LNCaP	Passages	Phosphatidylinositol 3‐kinase (PI3K)/Akt signaling pathway suppressed androgen receptor activity at passage 25, but enhanced androgen receptor activity at passage 60. High‐passage LNCaP cells displayed a considerably faster growth rate in the charcoal‐stripped serum medium than those at low passage	[52]
MIN‐6	Passages	The proliferation rate (doubling time) in high‐passage MIN‐6 cells is approximately two times faster than that of low‐passage MIN‐6 cells. mRNAs involved in secretion, adhesion and proliferation in MIN‐6 cells were expressed differently between passage 18 and passage 40. In high‐passage MIN‐6, 88 genes showed an at least twofold increase in expression, whereas 185 genes demonstrated a decrease in expression when compared to low‐passage MIN‐6	[53]
PMC42	Subclones	Comparative proteomics conducted on two PMC42 cell lines using mass spectrometry identified 244 differentially expressed proteins, with 73 upregulated and 61 downregulated in the epithelial PMC42‐LA cells. The differentially regulated proteins were found to be involved in various pathways such as glycolysis/gluconeogenesis, proteasome, protein processing in endoplasmic reticulum, and carbon metabolism	[54]

## 9 STRATEGIES TO MINIMIZE CIN DURING CELL CULTURE

Cell line instability has been a long‐standing issue highlighting concerns about data validity and reproducibility. While it is impossible to prevent a cell line from evolving in culture, researchers should take actions to improve the reliability and reproducibility of cell‐based research. Here we set out some recommendations for researchers to monitor and control CIN during cell culture.

1. The source of the cell lines should be carefully selected. Numerous academic or commercial organizations have established cell culture banks, such as American type culture collection (ATCC), Cellbank Australia, and Coriell cell repository.^1^ Cell lines from these sources are routinely tested for identity and potential microbial contamination, so they are unlikely to be misidentified or contaminated. However, it is a common practice for laboratories to obtain cell lines from their colleagues without verifying their condition. There is a tendency to assume that the cells will retain their properties regardless of their previous use. As a result, unstable or misidentified cell lines may be distributed around laboratories without anyone realizing the issue. These problematic cells may affect the experimental results in subsequent studies. Therefore, it is recommended that cell lines be obtained directly from ATCC or other cell banks to prevent quality issues from the beginning.
2. Frozen stocks of continuous cell lines should be produced at specific time points, so researchers can regularly return to these stocks to minimize the passage number or repeat experiments. Also, when a cell line is not used, it is preferable to return it to a cryopreserved bank to avoid unnecessary long‐term cell culture and passaging. When working with cell lines, it is recommended that primary tests be conducted to specify a passage number range that is appropriate for maintaining consistent cell functions that is, only a limited number of passages are performed before returning to another batch of the same stock. Importantly, since there is no easy way to determine how many times a cell culture has been passaged, every passaging must be properly recorded. When thawed from the freezer the passage number continues and is added to the number at freezing.
3. The growth environment of cell lines is a crucial factor that can significantly impact the stability of the cells. Even slight variations in the culture conditions can lead to significant changes in the characteristics of the cells. Frequent passages and transfers between laboratories can expose cells to varying environments, potentially leading to the selection of clones better adapted to the new conditions.^44^ To mitigate this risk, it is essential to carefully optimize and control all aspects of the cell cultural environment, including culture medium, passaging methods, temperature, humidity, and CO_2_ levels. Additionally, good laboratory practices are also critical to prevent contamination. Mycoplasma infection is a common contamination issue that can cause CIN by releasing enzymes that break down DNA, leading to mutations and chromosomal aberrations.^55^ Therefore, regular testing and monitoring of cell cultures for mycoplasma contamination are necessary.
4. Observing cell morphology under an inverted microscope is considered the simplest way to assess the quality and stability of cells. In general, cell lines with high CIN levels often undergo morphological changes and display heterogeneous cellular morphology.^1^ While such phenotype‐based methods are insufficient to determine the condition of cells, it is still recommended that researchers perform microscopy checks as a matter of course and keep digital records of the appearance of cells for comparisons.
5. Cell line authentication and characterization should be performed regularly using suitable methods, and baselines should be established to check for unacceptable changes throughout experiments. If certain cellular products or functions are being investigated, these characteristics should also be monitored to determine whether and how they change over time in culture. Currently, short tandem repeat (STR) profiling is widely used as a standard method for authenticating human cell lines because it is inexpensive and easy to interpret.^56^ Other cytogenetic techniques, including karyotyping and single nucleotide polymorphism analysis, may also be used. Each cell line has inherently different CIN levels, and the complex effects of CIN are dependent on various factors, such as the tissue of origin and the cultural conditions. Therefore, it is expected that all cultured cell lines should be routinely authenticated to ensure they remain genetically and phenotypically stable.

## 10 CONCLUSIONS AND PERSPECTIVES

As discussed in this review, CIN is a complex and multifaceted phenomenon that is caused by many different mechanisms and is regulated by hundreds of genes. Further research will help identify the central drivers and suppressors of CIN, as well as their interactions. Ultimately, a better understanding of CIN will enable us to prevent its associated issues during cell culture.

Current research on CIN is challenging due to the lack of a standardized definition and measurement criteria for CIN. Many studies based their estimates of CIN levels on their own data, and there is a lack of consensus about how to measure the level of CIN. Such ambiguity makes it difficult to interpret data and compare the results of different studies. CIN is defined as an increase in the rate of gain or loss of whole or fractions of chromosomes.^17^ Although related, aneuploidy and CIN are not synonyms. Aneuploidy refers to a condition characterized by an abnormal number of chromosomes, while CIN describes the ongoing process of changes in chromosome complements over time. Down syndrome (trisomy 21) is a well‐known example of cells displaying aneuploidy without CIN.

Not only do we need to clarify the definition of CIN, but we also need the appropriate techniques to measure it. The broad range of chromosomal abnormalities makes it extremely difficult to quantify CIN. Apart from common structural aberrations like translocations, duplications, deletions, and inversions, there are many unclassified aberrations that cannot be easily detected or quantified. One such chromosomal aberration is chromothripsis, a process of rapid and massive genome re‐organization that results in highly rearranged chromosomes.^49^ There is a great need to develop a standardized framework for CIN measurement, which necessitates clarification on what kinds of chromosome aberrations should be measured and how to quantify their contributions.

As discussed earlier, CIN is, by definition, the rate of gain or loss of fractional or whole chromosomes in a population of cells. Hence, the measurement of CIN requires monitoring of chromosomal changes over time. Such time‐based measurements are feasible on cell cultures but impossible on fixed tissue. Alternatively, CIN can be more readily assessed by measuring cell‐to‐cell variability at a single time point.^57^ Due to the intrinsic complexity of the time‐based approach, the single‐cell approaches are more frequently used as they can be easily applied to a wide range of samples, including clinical specimens. Numerous techniques have been developed to evaluate CIN at the single‐cell level, such as flow cytometry, fluorescent in situ hybridization (FISH), multiplex FISH (M‐FISH), and single‐cell sequencing.^57^ Each of these methods has its strengths and limitations. For example, M‐FISH is commonly used to visualize chromosomal aberrations because it can simultaneously paint all 24 different human chromosomes in different colors, enabling analysis of numerical aberrations and complex chromosomal rearrangements. However, M‐FISH fails to identify chromosomal abnormalities that do not lead to a noticeable color change, such as intrachromosomal inversions and small insertions and deletions below 3–5 Mb.^58^ The process also necessitates the use of dividing cells in metaphase, which can only be obtained through labour‐intensive cell culture techniques, considerably restricting its throughput. Hence, further experimental innovations are needed to allow rapid and accurate analysis of CIN within a cell population.

Understanding the consequences of chromosomal instability (CIN) in cancer cell lines is important for cancer research in several ways. The presence of CIN can be used as a diagnostic and prognostic marker for cancer and help identify subtypes of cancer that are more likely to have a poor prognosis or be resistant to treatment. Bold et al.^59^ look at the correlation between DNA damage response (DDR) during replication, chromosomal instability (CIN) score, and survival in head and neck squamous cell carcinoma (HNSCC) patients. They found that high CIN scores were associated with increased DDR during replication, and patients with high DDR had worse overall survival than those with low DDR. This suggests that DDR during replication and CIN score may be useful biomarkers for predicting survival in HNSCC patients and has implications for developing personalized treatment strategies for HNSCC based on CIN score and DDR during replication. CIN can contribute to drug resistance in cancer cells, making it a target for drug discovery and development. Sansregret et al.^60^ show that dysfunction of the Anaphase Promoting Complex/Cyclosome (APC/C), a protein complex involved in cell cycle regulation, can limit chromosomal instability (CIN) in cancer cells. The study suggests that APC/C dysfunction may be a mechanism through which cancer cells avoid excessive CIN and maintain genomic stability and may provide new therapeutic targets for cancer treatment.

CIN is a key driver of cancer development and progression. Understanding the mechanisms and consequences of CIN can help identify potential preventive strategies, such as lifestyle changes or targeted interventions to reduce the risk of cancer. Understanding the consequences of CIN in different cancer cell lines can help guide the development of personalized treatment strategies that consider the specific molecular characteristics of an individual's cancer. For example, Martín et al.^61^ identify mitochondrial RNA methyltransferase TRMT61B as a potential biomarker and therapeutic target for highly aneuploid cancers through an in‐silico search. TRMT61B is overexpressed in highly aneuploid cancers and its inhibition selectively induces cell death in these cancer cells. TRMT61B inhibition leads to a decrease in mitochondrial function, and the downregulation of genes involved in the regulation of the cell cycle and cell division. TRMT61B may serve as a new biomarker for highly aneuploid cancers and a potential therapeutic target selectively kill cancer cells with high levels of chromosomal instability. In summary, understanding the consequences of CIN in cancer cell lines is essential for developing effective strategies for cancer diagnosis, prevention, and treatment. It can also help identify new therapeutic targets and guide the development of personalized treatment plans for individual patients.

To conclude, cell lines remain a powerful tool for biomedical research, but the high level of CIN during culture and the variation across cell line strains must be well considered in experimental design and data interpretation. The scientific community need to work together to increase engagement and awareness. Organizations can formulate and refine the guidelines for the handling of cell lines. We, as responsible researchers, should take practical steps to address the issues arising from unstable cell lines and ensure the validity of our research.

## AUTHOR CONTRIBUTIONS

**Zichen He:** Investigation (lead); writing – original draft (lead). **Andrew David Wilson:** Formal analysis (supporting); project administration (supporting); supervision (supporting); writing – original draft (supporting). **Fenella Jean Rich:** Investigation (supporting); resources (supporting). **Diane Kenwright:** Investigation (supporting); resources (equal). **Aaron John Stevens:** Supervision (supporting); writing – review and editing (supporting). **Yee Syuen Low:** Investigation (supporting); project administration (supporting); resources (supporting). **Michelle Thunders:** Conceptualization (equal); funding acquisition (equal); project administration (equal); resources (equal); supervision (lead); writing – review and editing (equal).

## CONFLICT OF INTEREST STATEMENT

The authors have stated explicitly that there are no conflicts of interest in connection with this article.

## ETHICS STATEMENT

This is a review article and did not require consent or ethical approval.

## Data Availability

Data sharing not applicable to this article as no datasets were generated or analysed during the current study.
